# *Nelumbo nucifera* Petals Ameliorate Depressive-like Symptom and Cognitive Deficit in Unpredictable Chronic Mild Stress Mouse Model

**DOI:** 10.3390/nu17010094

**Published:** 2024-12-29

**Authors:** Juthamart Maneenet, Yutthana Chotritthirong, Ashraf M. Omar, Rattanathorn Choonong, Supawadee Daodee, Orawan Monthakantirat, Charinya Khamphukdee, Supaporn Pitiporn, Suresh Awale, Kinzo Matsumoto, Yaowared Chulikhit

**Affiliations:** 1 Division of Pharmaceutical Chemistry, Faculty of Pharmaceutical Sciences, Khon Kaen University, Khon Kaen 40002, Thailand; juthamart_pp@hotmail.com (J.M.); csupawad@kku.ac.th (S.D.); oramon@kku.ac.th (O.M.); 2 Natural Drug Discovery Laboratory, Institute of Natural Medicine, University of Toyama, 2630 Sugitani, Toyama 930-0194, Japan; ashraf.omar88@gmail.com (A.M.O.); suresh@inm.u-toyama.ac.jp (S.A.); 3 Graduate School of Pharmaceutical Sciences, Khon Kaen University, Khon Kaen 40002, Thailand; yutthana_ch@kkumail.com; 4 Department of Pharmacology, Faculty of Medicine, Khon Kaen University, Khon Kaen 40002, Thailand; rattach@kku.ac.th; 5 Division of Pharmacognosy and Toxicology, Faculty of Pharmaceutical Sciences, Khon Kaen University, Khon Kaen 40002, Thailand; charkh@kku.ac.th; 6 Department of Pharmacy, Chao Phya Abhaibhubejhr Hospital, Ministry of Public Health, Prachinburi 25000, Thailand; spitiporn@yahoo.com; 7 Graduate School of Pharmaceutical Sciences, Daiichi University of Pharmacy, Fukuoka 815-8511, Japan; k-matsumoto@daiichi-cps.ac.jp; 8 Division of Medicinal Pharmacology, Institute of Natural Medicine, University of Toyama, 2630 Sugitani, Toyama 930-0194, Japan

**Keywords:** *Nelumbo nucifera*, chronic mild stress, depression, anxiety, MAO inhibition, BDNF, CREB, SGK1

## Abstract

**Background** Chronic stress exposure has been widely recognized as a significant contributor to numerous central nervous system (CNS) disorders, leading to debilitating behavioral changes such as anxiety, depression, and cognitive impairments. The prolonged activation of the hypothalamic–pituitary–adrenal (HPA) axis during chronic stress disrupts the neuroendocrine balance and has detrimental effects on neuronal function and survival. *Nelumbo nucifera* (*N. nucifera*) Gaertn., commonly known as the lotus flower, is a traditional medicinal plant consumed for its purported benefits on mental and physical well-being. Despite its traditional use, limited scientific evidence supports these claims. **Methods** The present study explores the effects of *N. nucifera*, commonly known as the lotus flower, on cognitive performance and stress resilience in a mouse model subjected to unpredictable chronic mild stress (UCMS). **Results** Daily treatment significantly improved cognitive performance, alleviated depressive-like behaviors, and normalized hypothalamic–pituitary–adrenal (HPA) axis activity, as indicated by a 60.97% reduction in serum corticosterone. At the molecular level, *N. nucifera* petals also downregulated serum- and glucocorticoid-inducible kinase 1 (SGK1) mRNA expression while upregulating brain-derived neurotrophic factor (BDNF) mRNA expression and cyclic-adenosine monophosphate (cAMP) responsive element-binding protein (CREB) mRNA expression in the hippocampus and frontal cortex. These normalizations are critical, as chronic stress dysregulates HPA axis function, exacerbating behavioral changes. Furthermore, a phytochemical analysis resulted in the isolation of five major compounds, kaempferol (**1**), trifolin (**2**), kaempferol-3-neohesperidoside (**3**), icariside D_2_ (**4**), and β-sitosterol (**5**), each demonstrating significant monoamine oxidase (MAO) inhibitory activity. **Conclusions** These compelling findings suggest that *N. nucifera* petals not only alleviate stress-induced mood and cognitive deficits but also offer a promising avenue for modulating the HPA axis and promoting neuroprotection via essential neurotrophic factors and enzymatic pathways. We advocate for its potential as a complementary and alternative medicine for effective stress management. Future investigations should further explore its mechanisms of action and evaluate its clinical applicability in stress-related disorders.

## 1. Introduction

The coronavirus disease 2019 (COVID-19) pandemic caused an enormous rise in mental health problems worldwide [1,2,3]. Social distancing measures, quarantine requirements, and the stress associated with unemployment and potential COVID-19 infection are thought to be key contributors to this increase [3]. Long-term exposure to chronic stress is known to be detrimental to both mental and physical health and is associated with severe psychiatric disorders such as anxiety, depression, post-traumatic stress disorder (PTSD), and sleep disturbances [2]. Notably, chronic stress-induced depression is associated with attention deficits and impaired cognitive function [4,5]. The pathophysiology of depression is strongly connected to deficits in neurotrophins and dysfunction in the hypothalamic–pituitary–adrenal (HPA) axis. Chronic stress disrupts the negative feedback loop within the HPA axis, leading to adrenal hyperactivity and excessive glucocorticoid (GC) secretion [6,7]. Elevated GCs are associated with increased activity of serum- and glucocorticoid-inducible kinase 1 (SGK1) and its downstream signaling pathways [7,8,9]. Furthermore, depressed patients exhibit abnormally low levels of brain-derived neurotrophic factor (BDNF), a crucial regulator of neurogenesis and neuronal plasticity, contributing to hippocampal atrophy and neuronal loss [10,11]. The cyclic-adenosine monophosphate (cAMP) responsive element-binding protein (CREB) signaling pathway plays a vital role in regulating BDNF expression, and its upregulation may be essential for antidepressant treatment responses [12].

*Nelumbo nucifera* (*N. nucifera*), commonly known as the lotus flower, is an aquatic plant that has long been used medicinally in a variety of traditional systems, including Chinese, Indian, Thai, and Japanese medicine [13]. All parts of the lotus plant are edible and have been used to treat various ailments, including diarrhea, insomnia, body heat imbalance, gastritis, central nervous system disorders, and antipyretic effects [13,14,15]. Many studies reported the anti-obesity and anti-adipogenic effects of lotus leaf, seed, and root extract [16,17]. Based on the National List of Essential Medicine in Thailand, its stamens constitute the necessary ingredient in traditional Thai medicines for improving cardio-tonic and blood circulatory systems, as well as reducing blood glucose and blood lipid levels [18].

In addition, several pieces of evidence demonstrated that *N. nucifera* showed various neuropharmacological properties, such as cognition-enhancing effects [19], acetylcholinesterase inhibition [20], and anxiolytic and antidepressant activities [21]. Notably, *N. nucifera* flowers are rich in flavonoids known for their neuroprotective and neuropharmacological properties. It exhibited cognitive improvement in restraint stress rats by reducing brain oxidative damage and serum corticosterone levels [19]. Moreover, *N. nucifera* petal extract possessed multiple mechanisms of action against the AD pathological cascade, including anti-apoptosis and anti-Aβ aggregation [22].

Based on the established link between chronic stress and depression and the potential neuroprotective properties of *N. nucifera*, we hypothesized that *N. nucifera* petals might possess antidepressant and cognitive-enhancing effects. Therefore, this study aimed to investigate the efficacy of *N. nucifera* petal extract against chronic stress-induced depression and cognitive deficits in ICR mice while exploring the underlying mechanisms of action. Through phytochemical analysis, we sought to identify key bioactive compounds within *N. nucifera*, which may open avenues for further investigations into natural products offering alternative or complementary strategies to conventional therapeutic approaches. Ultimately, this research not only enhances our understanding of *N. nucifera*’s pharmacological potential but also paves the way for developing new interventions that can benefit diverse populations facing mental health challenges.

## 2. Materials and Methods

### 2.1. Plant Material

*N. nucifera* petals were collected in May 2019 from Bang Decha, Prachinburi, Thailand. The Chief of the Traditional Knowledge Center of Chao Phya Abhai Bhubejhr Hospital Foundation, Mrs. Benjawan Leenin, confirmed the botanical identification. A voucher specimen (ABH15) was deposited in the Chao Phya Abhai Bhubejhr Hospital Foundation’s herbarium.

### 2.2. General Experimental Procedures

NMR spectra were recorded using a JEOL ECX400 (Otemachi, Chiyoda, Tokyo, Japan), using CDCl_3_ as solvent. Medium-pressure liquid chromatography (MPLC) was performed with a Büchi MPLC C-605 double gradient pump system using normal-phase silica gel (silica gel 60 N, Kanto Chemical, Chuo-ku, Tokyo, Japan) and reverse-phase silica gel (Cosmosil 75C18-OPN, Nacalai Tesque, Inc., Kyoto, Japan). Analytical TLC was performed on pre-coated silica gel 60F_254_ and RP-18F_254_ plates (0.25 or 0.50 mm, Merck KGaA, Darmstadt, Germany). Preparative TLC was performed on pre-coated silica gel 70 PF254 plates (0.75 mm thickness) from Wako Pure Chemical Industries, Ltd., Chuo-ku, Osaka, Japan.

### 2.3. Extraction and Isolation

*N. nucifera* petals (2.8 kg) were extracted at room temperature with 95% EtOH (3 × 30 L, three days/cycle) [23]. The solvent was collected, evaporated at 50 °C under reduced pressure, and freeze-dried to obtain *N. nucifera* extract (300 g). This extract (300 g) was suspended in water and partitioned with EtOAc (5 × 500 mL) successively to give EtOAc (180 g) and H_2_O (120 g) soluble parts. The EtOAc soluble part was subjected to silica gel column chromatography using an acetone/CH_2_Cl_2_ solvent system gradient mixture (0–100%) followed by a MeOH/acetone gradient system (0–100%) to afford five fractions (Fr.1, 25.67 g; Fr.2, 17 g; Fr.3, 20.41 g; Fr.4, 14 g; Fr. 5, 85.33 g). Fraction 2 (17 g) was re-chromatographed on normal-phase MPLC using a n-hexane/acetone gradient mixture (0–100%) to give four subfractions (Fr.2-1, 7.68 g; Fr.2-2, 1.75 g; Fr.2-3, 3.89 g; Fr.2-4, 480 mg). Subfraction 2-2 was identified as β-sitosterol (**5**, 1.75 g). Subfraction 2–4 was re-chromatographed on silica gel with MPLC using a CH_2_Cl_2_/MeOH gradient system (0–100%) to afford kaempferol (**1**, 5.3 mg). Fraction 4 (14 g) was chromatographed over a reverse-phase silica gel column using an acetonitrile/water gradient system (0–100%) as a mobile phase to give trifolin (**2**, 2.55 g), kaempferol-1-3-neohesperidoside (**3**, 600 mg), and icariside D_2_ (**4**, 230 mg). The ^1^H NMR and ^13^C data are well-matched with the literature reported for compounds **1**–**5** [24,25,26,27,28].

### 2.4. Human Monoamine Oxidase (MAO) A/B Inhibition Assay

The inhibitory activity of the *N. nucifera* petal extract and its isolated compounds (**1**–**5**) against human recombinant MAO A/B was evaluated using a previously described method [29]. Stock solutions of the samples and positive controls (clorgyline for MAO-A and deprenyl for MAO-B) were prepared and serially diluted. The reaction mixture contained the enzyme, substrate (kynuramine), and test sample. Fluorescence spectrophotometry was used to measure the generated 4-hydroxyquinoline product at excitation and emission (λ_ex_ 310; λ_em_ 400 nm), respectively. The sample concentration required to inhibit 50% enzyme activity (IC_50_) was calculated from the generated sigmoidal dose–response curves using GraphPad Prism 5 software. The results are presented as mean ± standard deviation (SD).

### 2.5. Animals

Sixty male ICR mice, aged 4 weeks (Japan SLC Inc., Shizuoka, Japan), were utilized in this experiment. The University of Toyama Institutional Animal Use and Care Committee approved the experimental protocols (A2016INM-01/04/2016). All experiments were strictly implemented according to the Guiding Principles for the Care and Use of Animals (NIH Publications No. 80–23, revised in 2011) and complied with the ARRIVE guidelines 2.0. Animals were fed and watered freely in paper-bedded cages. The housing environments were a 12 h light–dark cycle (light on 07:00–19:00), a thermostatically controlled temperature of 24 ± 1 °C, and stable humidness (65 ± 2%). The behavioral tests occurred from 07:30 to 17:00 h.

### 2.6. Unpredictable Chronic Mild Stress (UCMS) Procedure

**Figure 1:**
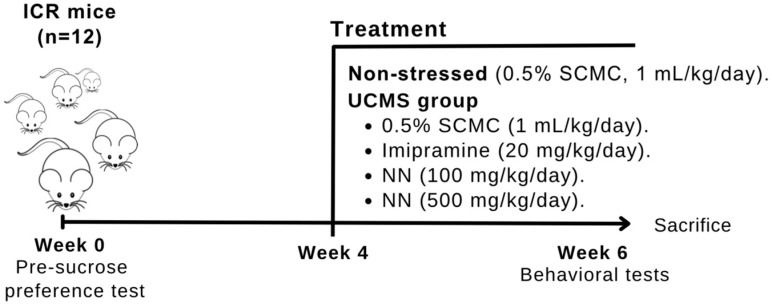
Schematic experiment protocols. Animals (*n* = 60) were divided into non-stressed and UCMS groups at week 0 (*n* = 12 mice per group). The UCMS groups received various stressors for 6 weeks. The UCMS mice were divided into four groups, which were daily administered with vehicle (0.5% SCMC/day), imipramine (20 mg/kg/day), and *Nelumbo nucifera* (NN) (100 and 500 mg/kg/day) for 3 weeks. In the sixth week, behavioral tests (modified Y-maze test, novel object recognition test, tail suspension test, and forced swimming test) were performed. After the behavioral tests, the animals were sacrificed to collect their blood and brains for neurochemical assessment.

Unpredictable chronic mild stress (UCMS) is a well-established animal model mimicking chronic stress and exhibiting features like human depression, with proven predictive face and construct validity [30]. This study employed the UCMS protocol outlined in Figure 1. Following a five-day acclimatization period in the animal room (week 0), the mice were randomly allocated to five groups based on their pre-sucrose preference test results: a non-stressed control and four UCMS groups. The non-stressed control group remained in their standard environment without additional stress exposure. UCMS groups were subjected to a variety of randomly scheduled stressors over six weeks, including food and water deprivation (18 h, 1 session), light exposure (36 h, 2 sessions), paired caging (2 h, 2 sessions), wet cage bedding (21 h, 1 session), tilted cage (45°, 12 h, 2 sessions), restricted food availability (5 pellets, 1 h, 2 sessions), intermittent white noise exposure (3 and 5 h, 2 sessions), and empty water bottle (3 h, 2 sessions). Sucrose preference tests were conducted weekly throughout the UCMS procedure to assess anhedonia, and additional behavioral tests were performed in week 6. Following the completion of UCMS, blood and brain samples were collected for neurochemical analysis (Figure 1).

### 2.7. Drug Administration

The vehicle used for the reference drug and the *N. nucifera* (NN) petals was 0.5% sodium carboxymethyl cellulose (SCMC). The reference drug, imipramine HCl, was administered intraperitoneally at 20 mg/kg. The NN was administered orally at 100 or 500 mg/kg doses. The reference drug and the extract were administered daily at 8:00 a.m., one hour before behavioral testing, starting in week 4 and continuing throughout the experiment. The non-stressed control group received the vehicle (0.5% SCMC) orally at 1 mL/kg.

### 2.8. Behavioral Studies

After five weeks of UCMS exposure, the mice were subjected to behavioral tests designed to assess their emotional and cognitive function. The tests were administered in a specific order, minimizing potential stress and carry-over effects, starting with the least stressful (modified Y-maze test) and progressing to more demanding tests: the novel object recognition test (NORT), tail suspension test (TST), and forced swimming test (FST). A two-day interval separated each test, and the mice only participated in each test once. During testing, the observers remained unaware of the experimental groups to minimize potential bias in their observations.

#### 2.8.1. Sucrose Preference Test (Anhedonia)

The sucrose preference test, conducted weekly throughout the experiment, as previously described [9], assessed anhedonia, a core symptom of depression. Mice were deprived of food and water for 18 h before the test to ensure motivation for fluid intake. On the test day, each mouse was randomized and individually housed in a cage, and two pre-weighed bottles were offered, one containing 2% (*w*/*v*) sucrose solution and the other containing water. After one hour, the bottles were removed, and their weight difference reflected the amount of each liquid consumed by the mouse. Sucrose intake was calculated using the following equation:

Sucrose intake (g/BW Kg) = amount of sucrose solution × 1000/body weight

#### 2.8.2. Modified Y-Maze Test

Spatial working memory was investigated using a modified Y-maze, as previously described [31]. It had three arms of equal length (40 cm long, 18 cm high, 3 cm wide at the bottom, and 12 cm wide at the top). This test consisted of two phases (sample and test phase trials). In the sample phase trial, the mice were individually located in the device with one of the three arms closed and allowed to freely explore the other two arms for 5 min. After 30 min, the mice explored for 5 min in the test phase trial with all three arms open. The duration of each arm’s exploration by the animals was timed and analyzed.

#### 2.8.3. Novel Object Recognition Test (NORT)

The NORT, a three-phase test used to assess short-term recognition memory in rodents, was performed as previously described [31]. During the habituation phase, the mice were allowed to freely explore an empty box (50 cm × 50 cm × 40 cm) for 15 min 24 h before testing. In the sample phase, the mice were individually placed in the box containing two identical objects for 5 min of exploration. Following a 30 min interval, the test phase began. One of the familiar objects was replaced with a novel object, and the mice were allowed to explore the box for another 5 min. The time spent exploring each object in both phases was recorded to calculate the discrimination index, which reflects recognition memory:

Discrimination index (%) = [(Time exploring novel object − Time exploring familiar object)/(Total exploration time)] × 100

A higher discrimination index indicates better learning and memory as mice explore the novel object more.

#### 2.8.4. Tail Suspension Test (TST)

The TST was conducted as previously described [9] to assess depression-like behavior in the mice. Briefly, the mice were individually suspended 60 cm above the floor by attaching an adhesive tape approximately 1 cm from the base of their tails. The duration of immobility during the last four minutes of a six-minute test session was measured. Mice in immobility were passively hanging without struggling. This test is based on the established principle that increased immobility during the TST reflects rodent depressive-like behavior [32].

#### 2.8.5. Forced Swimming Test (FST)

The forced swimming test (FST) is a widely used model for assessing depression-like behavior in rodents [33]. This test measures the time mice spend immobile when placed in an inescapable environment, such as a water-filled cylinder. The protocol followed established methods [34]. Briefly, the mice were individually placed in transparent glass cylinders (diameter 12 cm, height 25 cm) filled with water (depth 10 cm) maintained at 25 °C. During a pre-test phase 24 h before the test, the mice could swim for 15 min to adapt to the environment. One hour before the test, the mice received their respective drug treatments. The actual test session involved placing the mice back in the cylinders for 5 min, and the immobile time was recorded. 

### 2.9. Serum Corticosterone Levels

Following the completion of behavioral testing, the mice were euthanized under pentobarbital anesthesia (Nembutal^®^, 60 mg/kg, i.p.). Blood samples were collected via cardiac puncture and stored overnight at room temperature. The samples were centrifuged at 3000 rpm for 15 min at 4 °C the next day, and the supernatant was collected for examination. Serum corticosterone (CORT) levels were measured using an enzyme-linked immunosorbent assay (ELISA) kit from Assaypro LLC (St. Charles, MO, USA). In short, a 96-well microplate was filled with 25 µL of mouse serum and 25 µL of biotinylated corticosterone in each well. The plate was incubated for 2 h at room temperature with gentle shaking, followed by five washes with the provided wash buffer. Subsequently, 50 µL of streptavidin–peroxidase conjugate was added to each well and incubated for 30 min. The plate was washed with the wash buffer five times, followed by a 20 min incubation with 50 µL of chromogen substrate. Finally, the absorbance at 450 nm was measured immediately [34].

### 2.10. Quantitative Real-Time Polymerase Chain Reaction (qPCR)

The mRNA expression of BDNF, CREB, and SGK1 in the frontal cortex and hippocampus was examined using quantitative real-time PCR (qPCR). Following RNA extraction using TRIzol^®^ reagent, first-strand cDNA synthesis was performed with oligo (dT) primers and M-MLV reverse transcriptase. Specific primers were designed for each gene: β-actin (Forward: 5′-CAT CCG TAA AGA CCT CTA TGC CAA C-3′, Reverse: 5′-ATG GAG CCA CCG ATC CAC A-3′), BDNF (Forward: 5′-GAC AAG GCA ACT TGG CCT AC-3′, Reverse: 5′-CCT GTC ACA CAC GCT CAG CTC-3′), CREB (Forward: 5′-TAC CCA GGG AGG AGG AAT AC-3′, Reverse: 5′-GAG GCT GCT TGA ACA ACA AC-3′), and SGK1 (Forward: 5′-GGG TGC CAA GGA TGA CTT TA-3′, Reverse: 5′-CTC GGT AAA CTC GGG ATC GA-3′). β-actin was used as a housekeeping gene for normalization. The qPCR cycling conditions involved initial denaturation, followed by cycles of denaturation, annealing at specific temperatures (60 °C for β-actin, 57 °C for BDNF, and 58 °C for SGK1), and extension. Melting curve analysis ensured specific product formation and gene expression levels were calculated using the ΔΔCt method with β-actin as the reference [9].

### 2.11. Statistical Analysis

Statistical analyses were performed using SigmaStat^®^ version 3.5 software (SYSTAT variance Inc., Richmond, CA, USA). In vitro data are presented as mean ± standard deviation (SD), while in vivo data are expressed as mean ± standard error of the mean (SEM). The normality of the data was assessed to confirm whether the data met the required assumptions for parametric tests. If the assumptions of normal distribution were not satisfied, appropriate non-parametric tests, such as the Kruskal–Wallis test, were employed as alternatives. For comparisons between the non-stressed control and UCMS groups, paired Student *t*-tests were employed. For multiple comparisons, a one-way analysis of variance (ANOVA) followed by a post hoc Tukey’s test was utilized. Statistical significance was set at *p* < 0.05.

## 3. Results

### 3.1. Chemical Investigation of N. nucifera Petal Extract

**Figure 2:**
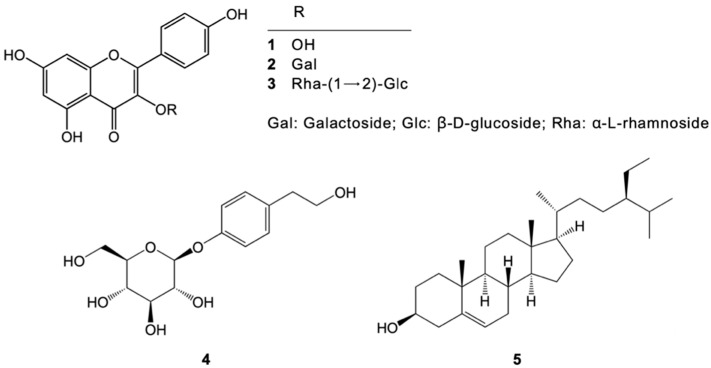
Structure of isolated compounds **1**–**5**.

The ethanolic extract of *N. nucifera* (NN) petals was fractionated using ethyl acetate (EtOAc) and water (H_2_O) to yield EtOAc- and H_2_O-soluble fractions. The EtOAc fraction was further purified using various chromatographic techniques (see the experimental description), leading to the isolation and identification of five compounds: kaempferol (1), trifolin (2), kaempferol-1-3-neohesperidoside (3), icariside D_2_ (4), and β-sitosterol (5). Nuclear magnetic resonance (NMR) spectroscopy was used to clarify the structures of these compounds (Figure 2). A detailed characterization of each compound, including NMR spectra and spectral data, is available in the Supplementary Materials.

### 3.2. Inhibitory Activity of N. nucifera Petal Extract and Its Constituents Against MAOs

The ethanolic extract of *N. nucifera* petals and its isolated constituents, kaempferol (**1**), trifolin (**2**), kaempferol-1-3-neohesperidoside (**3**), icariside D_2_ (**4**), and β-sitosterol (**5**), were evaluated for their inhibitory activity against monoamine oxidases (MAOs) A and B using recombinant human enzymes. Fluorescence spectrophotometry was used to measure MAO-mediated catalysis rates based on 4-hydroxyquinoline yield. All five compounds (**1**–**5**) demonstrated inhibitory effects against both MAO-A and MAO-B activity. Table 1 illustrates the half-maximal inhibitory concentration (IC_50_) values for each compound against both enzymes and the reference inhibitors deprenyl and clorgyline.

### 3.3. N. nucifera Petals (NN) Reverses UCMS-Induced Anhedonic Behavior in Mice

Anhedonia, a core symptom of depression, was assessed by measuring the difference in 2% sucrose consumption between the vehicle-treated UCMS group and the non-stressed control group. The mice subjected to the UCMS protocol displayed significantly reduced sucrose consumption compared to the control group from weeks 3 to 6 (*p* < 0.001). This finding confirms the establishment of anhedonia behavior in the UCMS model.

**Figure 3:**
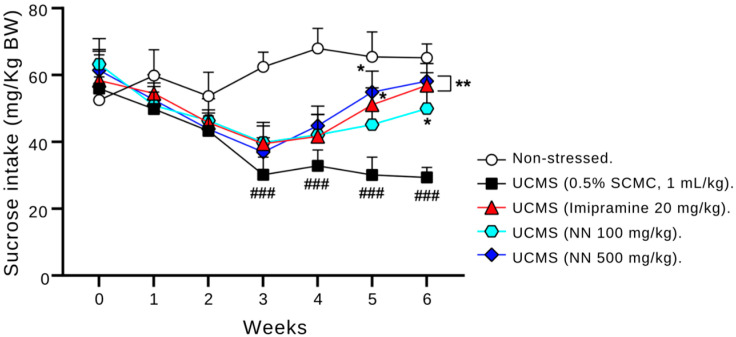
The effect of *N. nucifera* (NN) petals on anhedonia as measured by the sucrose preference test. After initiating the UCMS procedure, the amount of 2% sucrose consumed by each animal group was measured as an indicator of anhedonia behavior. Each line shows the mean ± S.E.M. (12 animals per group). ^###^ *p* < 0.001 vs. the non-stressed group. * *p* < 0.05 and ** *p* < 0.01 vs. the vehicle-treated UCMS group.

Treatment with the established antidepressant imipramine significantly increased sucrose consumption in the UCMS mice compared to the vehicle-treated group during weeks 5 and 6 (*p* < 0.05 and *p* < 0.001, respectively). Similarly, the oral administration of 500 mg/kg/day of NN significantly increased sucrose consumption in the UCMS mice in weeks 5 and 6 compared to the vehicle-treated group (*p* < 0.05 and *p* < 0.001, respectively) (Figure 3). These findings suggest that NN treatment may exert antidepressant-like effects, reversing UCMS-induced anhedonia behavior in mice to levels comparable to the non-stressed controls.

### 3.4. N. nucifera (NN) Petal Improves Memory in the UCMS Model

**Figure 4:**
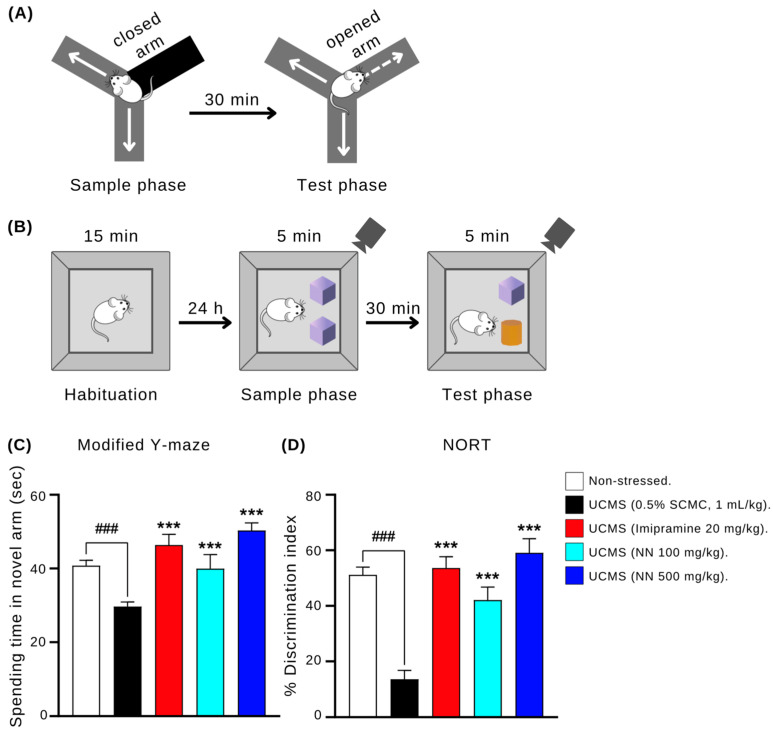
The effect of *N. nucifera* (NN) petals on cognitive function in the modified Y-maze test (**A**,**B**) and novel object recognition test (**C**,**D**). The time spent exploring the novel arm in the modified Y-maze test and the percentage of discrimination index of each animal group were measured 6 weeks after starting the UCMS procedure. Each column shows the mean ± S.E.M. (12 animals per group). ^###^ *p* < 0.001 vs. the non-stressed group. *** *p* < 0.001 vs. the vehicle-treated UCMS group.

The effects of NN petals on cognitive function in the UCMS mice were assessed using the modified Y-maze and novel object recognition (NORT) tests (Figure 4). These tests evaluate spatial and non-spatial working memory, respectively. Compared to the non-stressed controls, the UCMS mice treated with the vehicle spent significantly less time exploring the novel arm in the Y-maze test (*p* < 0.001), indicating impaired spatial working memory. Treatment with NN (500 mg/kg/day) significantly reversed this deficit, increasing exploration time in the novel arm compared to the vehicle-treated UCMS mice (*p* < 0.001). This effect was similar to that observed with the positive control, imipramine (*p* < 0.001). In the NORT, the mice spent equal time exploring identical objects during the sample phase. However, during the test phase, the UCMS mice spent less time exploring the novel object than the familiar object (*p* < 0.001), indicating impaired non-spatial working memory. Both imipramine (20 mg/kg/day) and NN (100 and 500 mg/kg/day) significantly improved discrimination between the novel and familiar objects compared to the vehicle-treated UCMS mice (*p* < 0.001) (Figure 4B). These findings suggest that NN treatment may improve both spatial and non-spatial working memory in the UCMS model, potentially alleviating cognitive dysfunction associated with depression.

### 3.5. N. nucifera (NN) Petals Reduce Hopelessness-like Behavior in the UCMS Model

**Figure 5:**
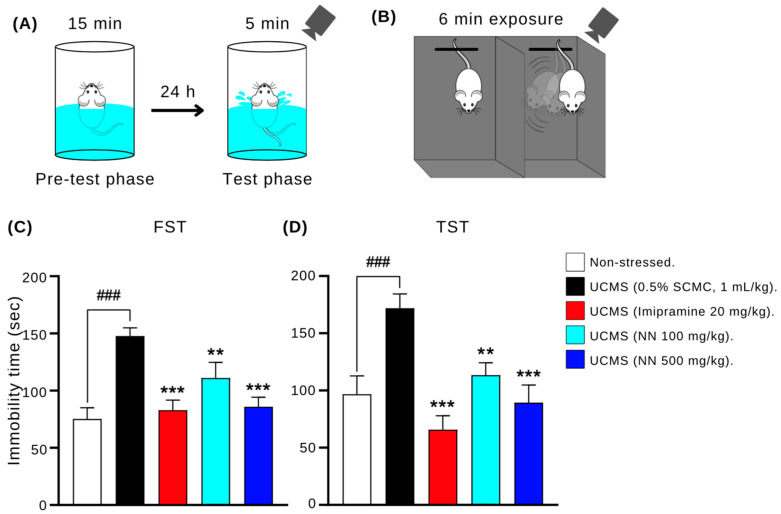
The effect of *N. nucifera* (NN) petals on hopeless behaviors in the forced swimming test (**A**,**B**) and tail suspension test (**C**,**D**). Six weeks after UCMS, each animal group’s immobility times were measured as an index of learned helplessness. Each column shows the mean ± S.E.M. (12 animals per group). ^###^ *p* < 0.001 vs. the non-stressed group. ** *p* < 0.01 and *** *p* < 0.001 vs. the vehicle-treated UCMS group.

The potential antidepressant-like effects of NN in the UCMS model of depression were assessed using the forced swimming test (FST) and tail suspension test (TST). Both tests measure immobility time, which serves as an indicator of learned helplessness behavior, a symptom commonly associated with depression. As shown in Figure 5, the vehicle-treated UCMS mice exhibited significantly higher immobility times than the non-stressed control group (*p* < 0.001). Conversely, the UCMS mice treated with either imipramine (20 mg/kg/day) or NN (500 mg/kg/day) displayed significantly reduced immobility times compared to the vehicle group (*p* < 0.001). These findings suggest that NN administration may exert an antidepressant-like effect by alleviating UCMS-induced hopelessness behavior.

### 3.6. Effects of N. nucifera (NN) Petals on Serum Corticosterone Levels

**Figure 6:**
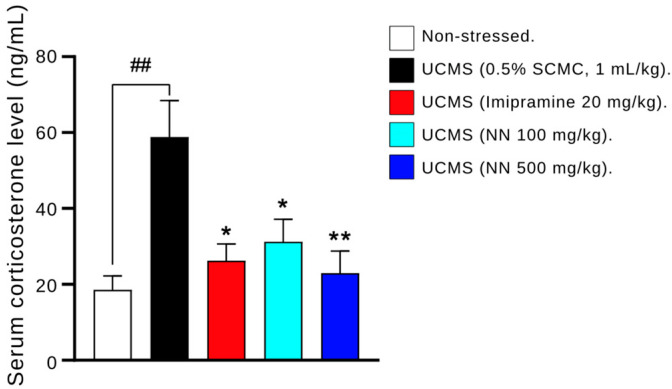
The effect of *N. nucifera* (NN) petals on serum corticosterone levels. Each column shows the mean ± S.E.M. (5 animals per group). ^##^ *p* < 0.01 vs. non-stressed group. * *p* < 0.05 and ** *p* < 0.01 vs. the vehicle-treated UCMS group.

The effect of UCMS on the hypothalamic–pituitary–adrenal (HPA) axis feedback mechanism was evaluated by measuring serum corticosterone (CORT) levels (Figure 6). As expected, UCMS significantly increased CORT levels compared to the non-stressed control group treated with the vehicle. Notably, the UCMS mice with NN at 100 and 500 mg/kg/day displayed significantly lower serum CORT levels than the vehicle-treated group (*p* < 0.05). This reduction in CORT levels by NN was comparable to the effect observed with the positive control, imipramine (*p* < 0.05). These findings suggest that NN may normalize HPA axis activity by reducing elevated corticosterone levels associated with UCMS.

### 3.7. N. nucifera (NN) Petals Modulate Gene Expression in the UCMS Model

**Figure 7:**
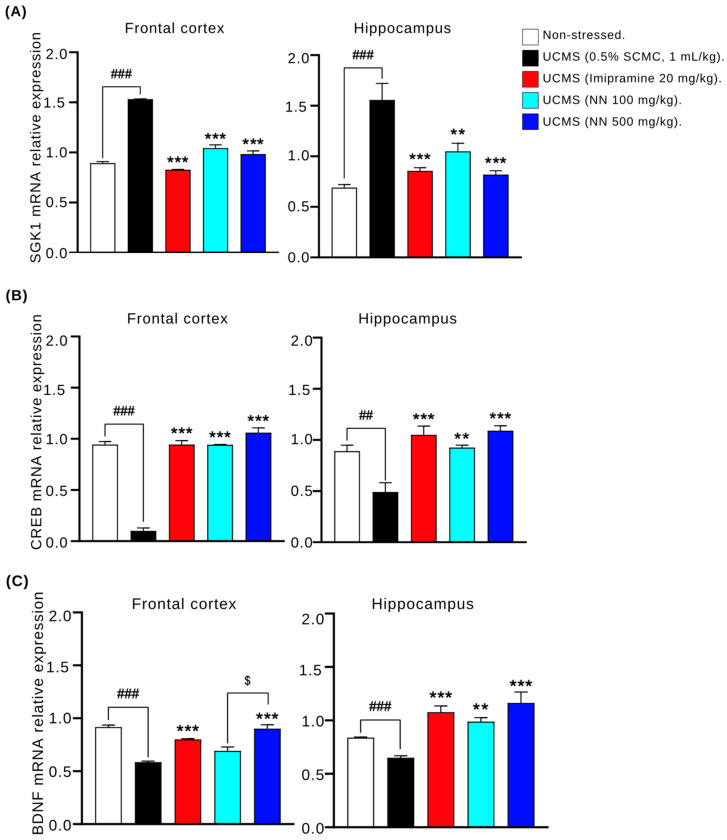
The effects of *N. nucifera* (NN) petals on the mRNA expression of SGK1 (**A**), CREB (**B**), and BDNF (**C**) in the frontal cortex and hippocampus. Each column shows the mean ± S.E.M. (5 animals per group). ^##^ *p* < 0.01 and ^###^ *p* < 0.001 vs. the vehicle-treated non-stressed group. ** *p* < 0.01 and *** *p* < 0.001 vs. the vehicle-treated UCMS group. ^$^ *p* < 0.05 compared to *N. nucifera* petal doses.

Quantitative real-time PCR (qPCR) analysis was performed on the frontal cortex and hippocampus tissues to evaluate the effects of NN on the expression of genes encoding SGK1, CREB, and BDNF. In both the frontal cortex and hippocampus, the UCMS group exhibited significantly increased SGK1 mRNA expression compared to the non-stressed controls (Figure 7A). Treatment with NN (100 and 500 mg/kg/day) or imipramine (20 mg/kg/day) significantly downregulated SGK1 mRNA expression in these brain regions, normalizing it to control levels. Conversely, the UCMS protocol caused a significant decrease in CREB and BDNF mRNA expression within the frontal cortex and hippocampus, as evident in Figure 7B and Figure 7C, respectively. This downregulation was reversed by the administration of both NN (100 and 500 mg/kg/day) and imipramine (20 mg/kg/day). Notably, NN exhibited a dose-dependent increase in hippocampal BDNF mRNA expression (Figure 7C). These findings suggest that NN may exert its antidepressant-like effects by modulating SGK1, CREB, and BDNF gene expression in the brain regions associated with mood regulation.

## 4. Discussion

The current study investigated the potential of *N. nucifera* (NN) petals to alleviate chronic unpredictable mild stress (UCMS)-induced cognitive deficits and depression-like behaviors in mice. Our findings demonstrate that NN treatment effectively improved both cognitive performance and depression-like behaviors compared to the UCMS controls. This suggests the potential benefits of NN in preventing the cognitive decline and depression associated with chronic stress. Phytochemical analysis of NN identified five major compounds: kaempferol (**1**), trifolin (**2**), kaempferol-1-3-neohesperidoside (**3**), icariside D2 (**4**), and β-sitosterol (**5**). Notably, kaempferol, a widely studied flavonoid class member, has documented neuroprotective and antitumor properties [35,36,37]. Additionally, research suggests that plant flavonoids, particularly kaempferol, exhibit antidepressant-like effects through their ability to inhibit MAO-A and MAO-B enzymes, which are implicated in the metabolism of mood-regulating neurotransmitters [38]. Animal models, like the well-established UCMS model, are instrumental in understanding the mechanisms underlying depression and cognitive dysfunction associated with chronic stress [39]. This model effectively replicates core depression symptoms in humans, including anhedonia, hopelessness, and learning and memory impairments, and these symptoms respond positively to antidepressant treatment [9,40].

Our study focused on the effects of *N. nucifera* (NN) petals on UCMS-induced depressive symptoms. Anhedonia, a hallmark symptom of depression, was evaluated through the sucrose preference test, where increased intake indicates reduced anhedonic behavior [9,40]. Notably, NN administration significantly increased sucrose consumption in the UCMS mice, mimicking the effect of the control drug, imipramine. Similarly, NN mirrored imipramine’s effect in the forced swim test (FST) and tail suspension test (TST) by significantly reducing immobility time, suggesting alleviation of hopelessness behavior [41,42]. These findings highlight the potential of NN in alleviating depression symptoms associated with chronic stress. There is strong evidence linking cognitive deficits in mild depressive disorder (MDD) to impairments in attention, learning, and memory, which can significantly disrupt daily functioning [5]. Antidepressant treatments often improve cognitive performance alongside mood elevation in depressive patients [5]. In this study, we evaluated the cognitive effects of NN using two memory paradigms: the modified Y-maze and the NORT. The modified Y-maze assessed short-term spatial working memory. Consistent with previous findings, the UCMS mice exhibited impaired performance, which was reversed by both imipramine and NN treatment. The NORT, which evaluates recognition memory based on preference for novel objects [43], further confirmed these results. The control mice demonstrated the expected preference for novel objects, whereas the UCMS mice showed no discrimination. Both NN and imipramine treatment restored object discrimination in the UCMS mice, indicating improved cognitive function.

The current study investigated the HPA axis, a key stress response system, as a potential mechanism underlying the observed antidepressant effects of NN. UCMS exposure, known to induce depression-like symptoms, triggered HPA axis hyperactivation, evident in elevated serum corticosterone levels. This finding aligns with previous research demonstrating a positive correlation between chronic stress and HPA axis dysregulation [9]. Further, consistent with these reports, we observed upregulated SGK1 expression, a protein mediating glucocorticoid effects and promoting glucocorticoid receptor (GR) activation. Cell stress and hormones, including glucocorticoids and mineralocorticoids, regulate SGK1 expression. Therefore, the putative role of SGK1 as a critical mediator in the dysregulation of the HPA axis is observed under chronic stress conditions [7,44]. Importantly, NN treatment mirrored the effects of the established antidepressant imipramine, significantly reducing serum corticosterone and downregulating SGK1 mRNA expression. This suggests that NN may exert its antidepressant-like effects, at least partially, by regulating the HPA axis. Furthermore, chronic stress and elevated glucocorticoid levels are associated with reduced hippocampal volume, impaired cognitive function, and dendritic atrophy in the CA3 region [45]. Notably, NN treatment effectively reversed UCMS-induced cognitive deficits, potentially suggesting a protective effect mediated by its influence on the HPA axis.

Recent preclinical and clinical studies have highlighted a possible link between chronic stress, cognitive decline, and the development of Alzheimer’s disease (AD) [46]. Chronic stress via dysregulation of the HPA axis can be a trigger of co-morbid depression in AD. Excessive corticosteroid secretion has been shown to hasten AD development in mice models, including intracellular tau hyperphosphorylation and extracellular beta-amyloid plaque deposition [46,47]. These extracellular beta-amyloid plaques and intracellular neurofibrillary tangles damage the healthy brain, such as the hippocampus and cerebral cortex, leading to neuronal loss and brain atrophy, which leads to AD [46]. In our previous study, results from in vitro assays revealed that NN petal extract effectively inhibited the aggregation of Aβ1-42, and this anti-aggregative activity demonstrated a strong correlation with the extract’s phenolic content. These findings significantly underscore the superior efficacy of *N. nucifera* in mitigating the effects of chronic stress exposure-induced cognitive decline with progression of AD [22].

The neurotrophic theory of depression highlights the critical role of neuronal plasticity in both disease development and antidepressant response [48]. BDNF, a crucial neurotrophic factor, supports cell survival, neurogenesis, and synaptic formation. Chronic stress suppresses BDNF mRNA expression via various signaling pathways, including MAPK, PLC-g, and PI3K [49], leading to the disruption of neuronal plasticity, neurogenesis impairment, and neuronal atrophy [50]. Ali S.S. and co-workers found chronic stress-induced hippocampal neuron atrophy, apoptosis, and decreased glial fibrillary acidic protein (GFAP)-positive cells and BDNF immune expression in the hippocampus [51]. Consequently, reductions in BDNF levels are associated with cellular atrophy in the prefrontal cortex and hippocampus—areas strongly linked to depression [51,52]. Additionally, the CREB-BDNF signaling pathway is vital for neuronal survival, synaptic structure, and plasticity, and antidepressant treatment promotes these events by regulating BDNF and CREB gene expression in the hippocampus of chronically stressed mice [53,54,55]. Consistent with these concepts, our study revealed that the UCMS paradigm reduced CREB and BDNF mRNA expression in the mice’s hippocampus and frontal cortex. Treatment with NN and the control drug imipramine significantly counteracted this UCMS-induced downregulation of BDNF and CREB mRNA [56]. Since BDNF supports memory formation and is often reduced in cognitive decline and depression [51,57], our findings suggest that its upregulation by NN may be one of the plausible mechanisms for improving UCMS-induced cognitive impairment and depression.

The monoamine oxidase (MAO) hypothesis suggests that MAO-A and MAO-B enzymes contribute to depression by metabolizing mood-regulating neurotransmitters like serotonin, norepinephrine, and dopamine [58,59]. This study investigated the potential MAO inhibitory activity of NN and its isolated compounds kaempferol, trifolin, kaempferol-1-3-neohesperidoside, icariside D_2_, and β-sitosterol. In vitro experiments revealed that NN and its identified compounds significantly inhibited MAO-A and MAO-B, suggesting a potential additional mechanism for NN’s antidepressant-like effects.

In summary, our findings demonstrate that NN petals exhibit potent antidepressant properties and effectively improve cognitive impairments associated with chronic stress. The findings indicate that *N. nucifera* not only alleviates symptoms of depression but also enhances memory and cognitive function, presenting a dual benefit that is crucial for individuals facing stress-related disorders. Given the alarming prevalence of co-symptoms such as dementia and depression, particularly in populations exposed to chronic stress, the ability of lotus petals to address both mood and cognitive deficits is especially significant. This superior effect positions *N. nucifera* as a promising candidate for therapeutic use, offering a natural alternative or complement to conventional pharmacological treatments. Nevertheless, few animals and no human clinical trial studies have investigated the beneficial effects of *N. nucifera* petals on AD. Therefore, further pharmacokinetic and toxicity research is necessary to validate their therapeutic efficacy and potential adverse effects in the treatment of stress-related conditions.

## 5. Conclusions

This study provides compelling evidence for the potential of *N. nucifera* petals as a therapeutic intervention for chronic stress-induced depression and cognitive dysfunction. *N. nucifera* petals demonstrated potent vitro MAO-A and MAO-B inhibition, suggesting a potential biochemical mechanism for its observed antidepressant-like effects. In vivo, NN treatment in the UCMS model effectively reversed behavioral deficits, including anhedonia, hopelessness, and cognitive impairments, mirroring the effects of the established antidepressant imipramine. Furthermore, NN treatment normalized HPA axis activity by reducing serum corticosterone and SGK1 expression while promoting neurogenesis and neuroplasticity through increased BDNF and CREB expression. These findings suggest that NN may provide benefits through a multifaceted approach, targeting neurotransmitter metabolism and key signaling pathways involved in mood regulation, neuronal survival, and cognitive function.

Identifying five major phytochemicals in NN extract, including kaempferol, provides a basis for further investigation into the specific bioactive components responsible for its observed effects. Overall, our research provides valuable insights into the molecular mechanisms underpinning the impact of *N. nucifera*. By harnessing the unique properties of this traditional medicinal plant, we may pave the way for innovative strategies that promote mental well-being, cognitive resilience, and comprehensive treatment for those affected by chronic stress, depression, and dementia.

## Figures and Tables

**Table 1:** Inhibitory effect of isolated compounds **1**–**5** on monoamine oxidase activities.

Compound	IC_50_ (µM)	
	MAO-A	MAO-B
*N. nucifera*	129.6 ± 1.98	387.5 ± 2.05
**1**	10.2 ± 0.23	93.5 ± 0.9
**2**	578.4 ± 2.01	860.7± 3.7
**3**	108.9 ± 0.21	242.2 ± 0.78
**4**	583.7 ± 11.8	ND ^1^
**5**	2.8 ± 0.13	ND ^2^
Clorgyline	0.014 ± 0.0001	3.88 ± 0.05
Deprenyl	35.13 ± 0.96	0.156 ± 0.02

^1^ Percentage of inhibition of MAO-B at concentration 1000 µM is 43.5 ± 5.2. ^2^ Percentage of inhibition of MAO-B at concentration 1000 µM is 20.7 ± 1.8.

## Data Availability

The original contributions presented in this study are included in the article/Supplementary Material. Further inquiries can be directed to the corresponding author.

## Supplementary Materials

The following supplementary materials are available for download at www.mdpi.com/10.3390/nu17010094/s1. Table S1. Sucrose preference test (one-way ANOVA), Table S2. Modified Y-maze test (one-way ANOVA), Table S3. Novel object recognition test (one-way ANOVA), Table S4. Forced swimming test (one-way ANOVA), Table S5. Tail suspension test (one-way ANOVA), Table S6. Serum corticosterone level (one-way ANOVA), Table S7. Brain-derived neurotrophic factor (BDNF) mRNA expression (one-way ANOVA), Table S8. Cyclic-adenosine monophosphate (cAMP) responsive element-binding protein (CREB) mRNA expression (one-way ANOVA), Table S9. Serum- and glucocorticoid-inducible kinase 1 (SGK1) mRNA expression (one-way ANOVA), Figure S1. ^1^H NMR spectrum of kaempferol (**1**) (400 MHz, CD_3_OD), Table S10. ^1^H NMR spectroscopic data of **1** in CD_3_OD (*δ* in ppm, *J* in Hz), Figure S2. ^1^H NMR spectrum of trifolin (**2**) (400 MHz, CDCl_3_), Figure S3. ^13^C NMR spectrum of trifolin (**2**) (100 MHz, CDCl_3_), Table S11. ^1^H and ^13^C NMR spectroscopic data of **2** in CDCl_3_ (*δ* in ppm, J in Hz), Figure S4. ^1^H NMR spectrum of kaempferol-3-neohesperidoside (**3**) (400 MHz, CD_3_OD), Figure S5. ^13^C NMR spectrum of kaempferol-3-neohesperidoside (**3**) (100 MHz, CD_3_OD), Table S12. ^1^H and ^13^C NMR spectroscopic data of **3** in CD_3_OD (*δ* in ppm, *J* in Hz), Figure S6. ^1^H NMR spectrum of icariside D_2_ (**4**) (400 MHz, CD_3_OD), Figure S7. ^13^C NMR spectrum of icariside D_2_ (**4**) (100 MHz, CD_3_OD), Table S13. ^1^H and ^13^C NMR spectroscopic data of **4** in CD_3_OD (*δ* in ppm, *J* in Hz), Figure S8. ^1^H NMR spectrum of β-sitosterol (**5**) (400 MHz, CDCl_3_), Figure S9. ^13^C NMR spectrum of β-sitosterol (**5**) (100 MHz, CDCl_3_), Table S14. ^1^H and ^13^C NMR spectroscopic data of **5** in CDCl_3_ (*δ* in ppm, *J* in Hz).
