# Supplementary material for: Nelumbo nucifera Petals Ameliorate Depressive-like Symptom and Cognitive Deficit in Unpredictable Chronic Mild Stress Mouse Model
Source: Nutrients. 2024 Dec 29;17(1):94. doi: 10.3390/nu17010094 (PMC11723358; doi:10.3390/nu17010094)
Supplement: Supplementary file 1 [file nutrients-17-00094-s001.zip › nutrients-3385096-supplementary.pdf]

Supplementary Materials

Nelumbo nucifera Petals Ameliorate Depressive-like Symptom and Cognitive Deficit in Unpredictable Chronic Mild Stress Mouse Model

Juthamart Maneenet [1,2], Yutthana Chotritthirong [3], Ashraf M. Omar [2], Rattanathorn Choonong [4], Supawadee Daodee [1], Orawan Monthakantirat [1], Charinya Khamphukdee [5], Supaporn Pitiporn [6], Suresh Awale [2], Kinzo Matsumoto [7,8] and Yaowared Chulikhit [1,*]

[1] Division of Pharmaceutical Chemistry, Faculty of Pharmaceutical Sciences, Khon Kaen University, Khon Kaen 40002, Thailand; juthamart_pp@hotmail.com (J.M.); csupawad@kku.ac.th (S.D.); oramon@kku.ac.th (O.M.); yaosum@kku.ac.th (Y.C.)

[2] Natural Drug Discovery Laboratory, Institute of Natural Medicine, University of Toyama, 2630 Sugitani, Toyama 930-0194, Japan; ashraf.omar88@gmail.com (A.M.O.); suresh@inm.u-toyama.ac.jp (S.A.)

[3] Graduate School of Pharmaceutical Sciences, Khon Kaen University, Khon Kaen 40002, Thailand; yutthana_ch@kkumail.com

[4] Department of Pharmacology, Faculty of Medicine, Khon Kaen University, Khon Kaen 40002, Thailand; rattach@kku.ac.th

[5] Division of Pharmacognosy and Toxicology, Faculty of Pharmaceutical Sciences, Khon Kaen University, Khon Kaen 40002, Thailand; charkh@kku.ac.th

[6] Department of Pharmacy, Chao Phya Abhaibhubejhr Hospital, Ministry of Public Health, Prachinburi 25000, Thailand; spitiporn@yahoo.com

[7] Graduate School of Pharmaceutical Sciences, Daiichi University of Pharmacy, Fukuoka 815-8511, Japan; k-matsumoto@daiichi-cps.ac.jp

[8] Division of Medicinal Pharmacology, Institute of Natural Medicine, University of Toyama, 2630 Sugitani, Toyama 930-0194, Japan

* Correspondence: yaosum@kku.ac.th; Tel.: +66-81-3802357

1. Statistical analysis of effects of *N. nucifera* petals extract (NN) on UCMS–induced anhedonia behavior by using sucrose preference test

Table S1. Paired Student's *t-test* and One-way analysis of variance (ANOVA) test of sucrose preference test.

Group comparison	ANOVA followed by Tukey's post hoc test	
	P	F(DF$_{between\ group}$, DF$_{residual}$)
**Week 0**		
non-stress group vs. UCMS + vehicle-treated group	0.811	T(22) = -0.241
All UCMS-induced groups	0.995	F(3,44) = 0.0236
**Week 1**		
non-stress group vs. UCMS + vehicle-treated group	0.291	t(22) = 1.081
All UCMS-induced groups	0.931	F(3,43) = 0.147
**Week 2**		
non-stress group vs. UCMS + vehicle-treated group	0.217	t(20) = 1.275
All UCMS-induced groups	0.964	F(3,43) = 0.0913
**Week 3**		
non-stress group vs. UCMS + vehicle-treated group	<0.001	t(19) = 4.740
All UCMS-induced groups	0.545	F(3,36) = 0.723
**Week 4**		
non-stress group vs. UCMS + vehicle-treated group	<0.001	t(22) = 4.588
All UCMS-induced groups	0.499	F(3,43) = 0.803
**Week 5**		
non-stress group vs. UCMS + vehicle-treated group	<0.001	t(22) = 3.859
All UCMS-induced groups	0.011	F(3,43) = 4.194
UCMS + vehicle-treated group vs. UCMS + IMP20 group	0.038	
UCMS + vehicle-treated group vs. UCMS + NN100 group	0.221	
UCMS + vehicle-treated group vs. UCMS + NN500 group	0.01	
UCMS + NN100 group vs. UCMS + NN500 group	0.591	
**Week 6**		
non-stress group vs. UCMS + vehicle-treated group	<0.001	t(22) = 7.013
All UCMS-induced groups	<0.001	F(3,43) = 9.113
UCMS + vehicle-treated group vs. UCMS + IMP20 group	<0.001	
UCMS + vehicle-treated group vs. UCMS + NN100 group	0.012	
UCMS + vehicle-treated group vs. UCMS + NN500 group	<0.001	
UCMS + NN100 group vs. UCMS + NN500 group	0.576	

2. Statistical analysis of effects of *N. nucifera* petals extract (NN) on UCMS-induced cognitive dysfunction by using modified Y-maze test and novel object recognition test

Table S2. Paired Student's *t-test* and One-way analysis of variance (ANOVA) test of modified Y-maze test

**Group comparison**	**ANOVA followed by Tukey's post hoc test**	
	P	$F(DF_{between\ group}, DF_{residual})$
non-stress group vs. UCMS + vehicle-treated group	<0.001	t(22) = 5.836
All UCMS-induced groups	<0.001	F(3,43) = 18.183
UCMS + vehicle-treated group vs. UCMS + IMP20 group	<0.001	
UCMS + vehicle-treated group vs. UCMS + NN100 group	<0.001	
UCMS + vehicle-treated group vs. UCMS + NN500 group	<0.001	
UCMS + NN100 group vs. UCMS + NN500 group	0.174	

Table S3. Paired Student's *t-test* and One-way analysis of variance (ANOVA) test of novel object recognition test

**Group comparison**	**ANOVA followed by Tukey's post hoc test**	
	P	$F(DF_{between\ group}, DF_{residual})$
non-stress group vs. UCMS + vehicle-treated group	<0.001	t(22) = 9.765
All UCMS-induced groups	<0.001	F(3,43) = 24.929
UCMS + vehicle-treated group vs. UCMS + IMP20 group	<0.001	
UCMS + vehicle-treated group vs. UCMS + NN100 group	<0.001	
UCMS + vehicle-treated group vs. UCMS + NN500 group	<0.001	
UCMS + NN100 group vs. UCMS + NN500 group	0.102	

3. Statistical analysis of effects of *N. nucifera* petals extract (NN) on UCMS-induced hopeless behaviors using forced swimming test and tail suspension test

Table S4. Paired Student's *t-test* and One-way analysis of variance (ANOVA) test of forced swimming test

Group comparison	ANOVA followed by Tukey's post hoc test	
	P	F($DF_{between\ group}$, $DF_{residual}$)
non-stress group vs. UCMS + vehicle-treated group	<0.001	t(21) = -5.941
All UCMS-induced groups	<0.001	F(3,42) = 11.044
UCMS + vehicle-treated group vs. UCMS + IMP20 group	<0.001	
UCMS + vehicle-treated group vs. UCMS + NN100 group	0.002	
UCMS + vehicle-treated group vs. UCMS + NN500 group	<0.001	
UCMS + NN100 group vs. UCMS + NE500 group	0.855	

Table S5. Paired Student's *t-test* and One-way analysis of variance (ANOVA) test of tail suspension test

Group comparison	ANOVA followed by Tukey's post hoc test	
	P	F($DF_{between\ group}$, $DF_{residual}$)
non-stress group vs. UCMS + vehicle-treated group	<0.001	t(22) = -4.179
All UCMS-induced groups	<0.001	F(3,43) = 18.185
UCMS + vehicle-treated group vs. UCMS + IMP20 group	<0.001	
UCMS + vehicle-treated group vs. UCMS + NN100 group	0.001	
UCMS + vehicle-treated group vs. UCMS + NN500 group	<0.001	
UCMS + NN100 group vs. UCMS + NN500 group	0.775	

4. Statistical analysis of effects of *N. nucifera* petals extract (NN) on UCMS-induced HPA-axis via serum corticosterone level

Table S6. Paired Student's *t-test* and One-way analysis of variance (ANOVA) test of serum corticosterone level

Group comparison	ANOVA followed by Tukey's post hoc test	
	P	F($DF_{between\ group}$, $DF_{residual}$)
non-stress group vs. UCMS + vehicle-treated group	0.005	t(8) = -3.895
All UCMS-induced groups	0.007	F(3,16) = 5.895
UCMS + vehicle-treated group vs. UCMS + IMP20 group	0.017	
UCMS + vehicle-treated group vs. UCMS + NN100 group	0.047	
UCMS + vehicle-treated group vs. UCMS + NN500 group	0.008	
UCMS + NN100 group vs. UCMS + NN500 group	0.82	

5. Statistical analysis of effects of *N. nucifera* petals extract (NN) on UCMS-induced dysfunction of BDNF, CREB and SGK1 mRNAs expression by using quantitative real-time polymerase chain reaction (QPCR) in frontal cortex and hippocampus

Table S7. Paired Student's *t-test* and One-way analysis of variance (ANOVA) test of brain-derived neurotrophic factor (BDNF) mRNA expression in frontal cortex and hippocampus

Group comparison	ANOVA followed by Tukey's post hoc test	
	P	$F(DF_{between\ group}, DF_{residual})$
**Frontal cortex**		
non-stress group vs. UCMS + vehicle-treated group	<0.001	t(8) = 16.299
All UCMS-induced groups	<0.001	F(3,16) = 26.922
UCMS + vehicle-treated group vs. UCMS + IMP20 group	<0.001	
UCMS + vehicle-treated group vs. UCMS + NN100 group	0.051	
UCMS + vehicle-treated group vs. UCMS + NN500 group	<0.001	
UCMS + NN100 group vs. UCMS + NN500 group	<0.001	
**Hippocampus**		
non-stress group vs. UCMS + vehicle-treated group	<0.001	t(8) = 10.063
All UCMS-induced groups	<0.001	F(3,16) = 13.385
UCMS + vehicle-treated group vs. UCMS + IMP20 group	<0.001	
UCMS + vehicle-treated group vs. UCMS + NN100 group	0.006	
UCMS + vehicle-treated group vs. UCMS + NN500 group	<0.001	
UCMS + NN100 group vs. UCMS + NN500 group	0.225	

Table S8. Paired Student's *t-test* and One-way analysis of variance (ANOVA) test of cAMP-response element binding protein (CREB) mRNA expression in frontal cortex and hippocampus

Group comparison	ANOVA followed by Tukey's post hoc test	
	P	$F(DF_{between\ group}, DF_{residual})$
**Frontal cortex**		
non-stress group vs. UCMS + vehicle-treated group	<0.001	t(8) = 20.774
All UCMS-induced groups	<0.001	F(3,16) = 166.421
UCMS + vehicle-treated group vs. UCMS + IMP20 group	<0.001	
UCMS + vehicle-treated group vs. UCMS + NN100 group	<0.001	
UCMS + vehicle-treated group vs. UCMS + NN500 group	<0.001	
UCMS + NN100 group vs. UCMS + NN500 group	0.112	
**Hippocampus**		
non-stress group vs. UCMS + vehicle-treated group	0.006	t(8) = 3.662
All UCMS-induced groups	<0.001	F(3,16) = 16.248
UCMS + vehicle-treated group vs. UCMS + IMP20 group	<0.001	
UCMS + vehicle-treated group vs. UCMS + NN100 group	0.002	
UCMS + vehicle-treated group vs. UCMS + NN500 group	<0.001	
UCMS + NN100 group vs. UCMS + NN500 group	0.351	

Table S9. Paired Student's *t-test* and One-way analysis of variance (ANOVA) test of serum- and glucocorticoid-inducible kinase 1 (SGK1) mRNA expression in frontal cortex and hippocampus

**Group comparison**	**ANOVA followed by Tukey's post hoc test**	
	P	$F(DF_{between\ group}, DF_{residual})$
**Frontal cortex**		
non-stress group vs. UCMS + vehicle-treated group	<0.001	t(8) = -39.861
All UCMS-induced groups	<0.001	F(3,16) = 360.863
UCMS + vehicle-treated group vs. UCMS + IMP20 group	<0.001	
UCMS + vehicle-treated group vs. UCMS + NN100 group	<0.001	
UCMS + vehicle-treated group vs. UCMS + NN500 group	<0.001	
UCMS + NN100 group vs. UCMS + NN500 group	0.218	
**Hippocampus**		
non-stress group vs. UCMS + vehicle-treated group	<0.001	t(8) = -5.226
All UCMS-induced groups	<0.001	F(3,16) = 13.09
UCMS + vehicle-treated group vs. UCMS + IMP20 group	<0.001	
UCMS + vehicle-treated group vs. UCMS + NN100 group	0.007	
UCMS + vehicle-treated group vs. UCMS + NN500 group	<0.001	
UCMS + NN100 group vs. UCMS + NN500 group	0.341	

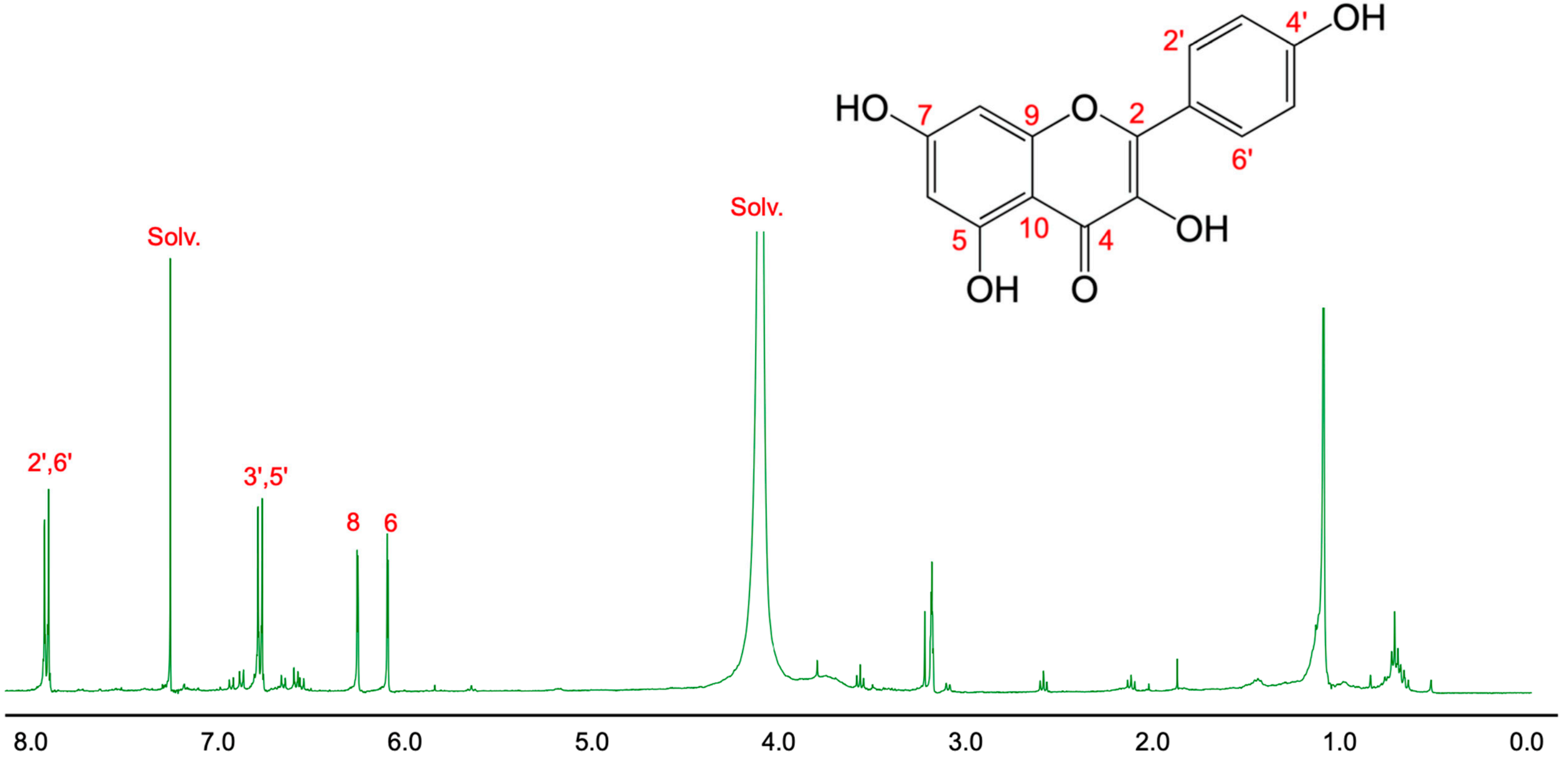

Figure S1. ^{1}H NMR spectrum of kaempferol (**1**) (400 MHz, CD_3OD).

Table S10. ^{1}H spectroscopic data of **1** in CD_3OD (δ in ppm, J in Hz).

no.	**1**
	δ_H
1	-
2	-
3	-
4	-
5	-
6	6.09, d, J=1.8
7	-
8	6.25, d, J=1.8
9	-
10	-
1'	-
2'	7.91, d, J=8.5
3'	6.77, d, J=8.5
4'	-
5'	6.77, d, J=8.5
6'	7.91, d, J=8.5
1''	-
2''	-
3''	-
4''	-
5''	-
6''	-
1'''	-
2'''	-
3'''	-
4'''	-
5'''	-
6'''	-

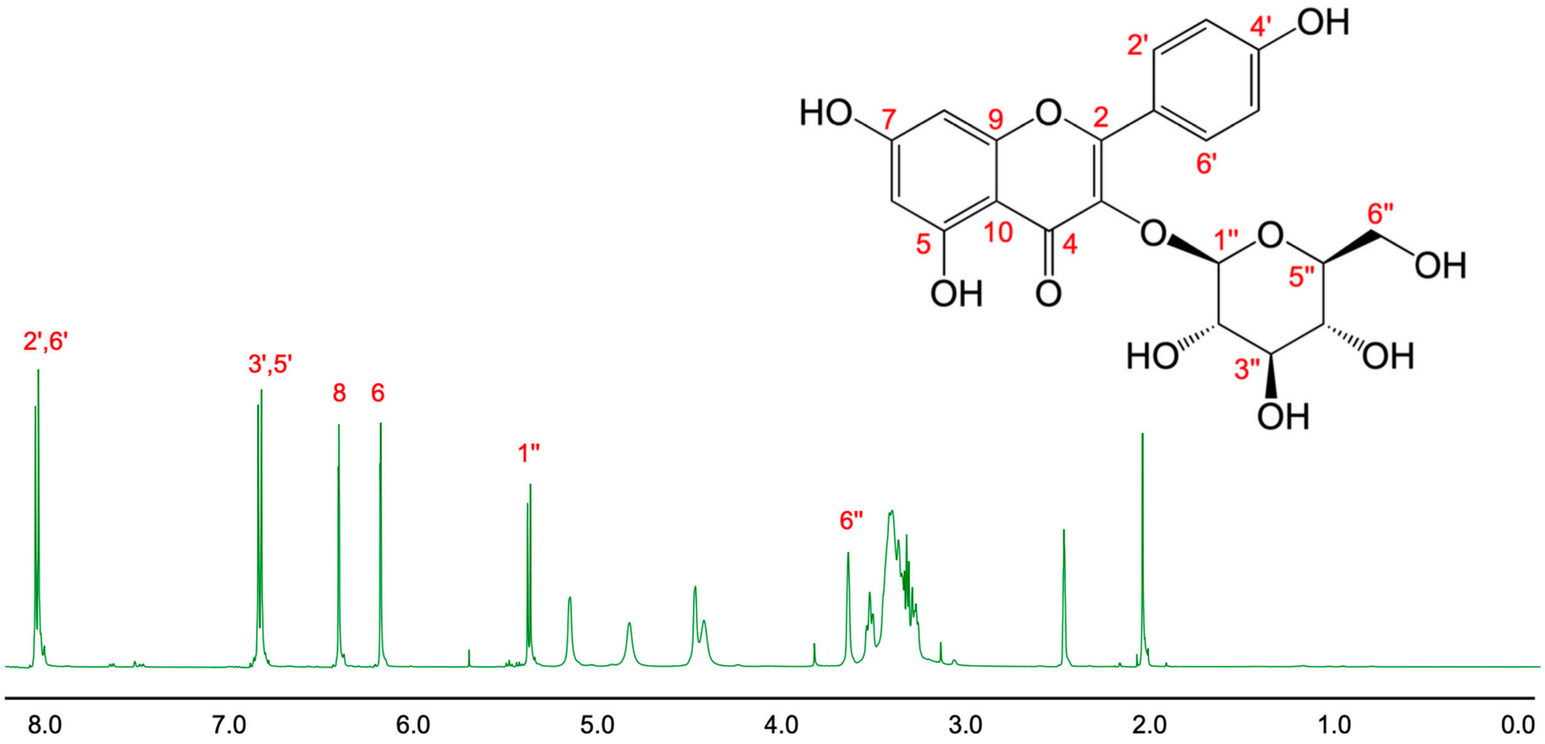

Figure S2. ^{1}H NMR spectrum of trifolin (**2**) (400 MHz, $CDCl_3$).

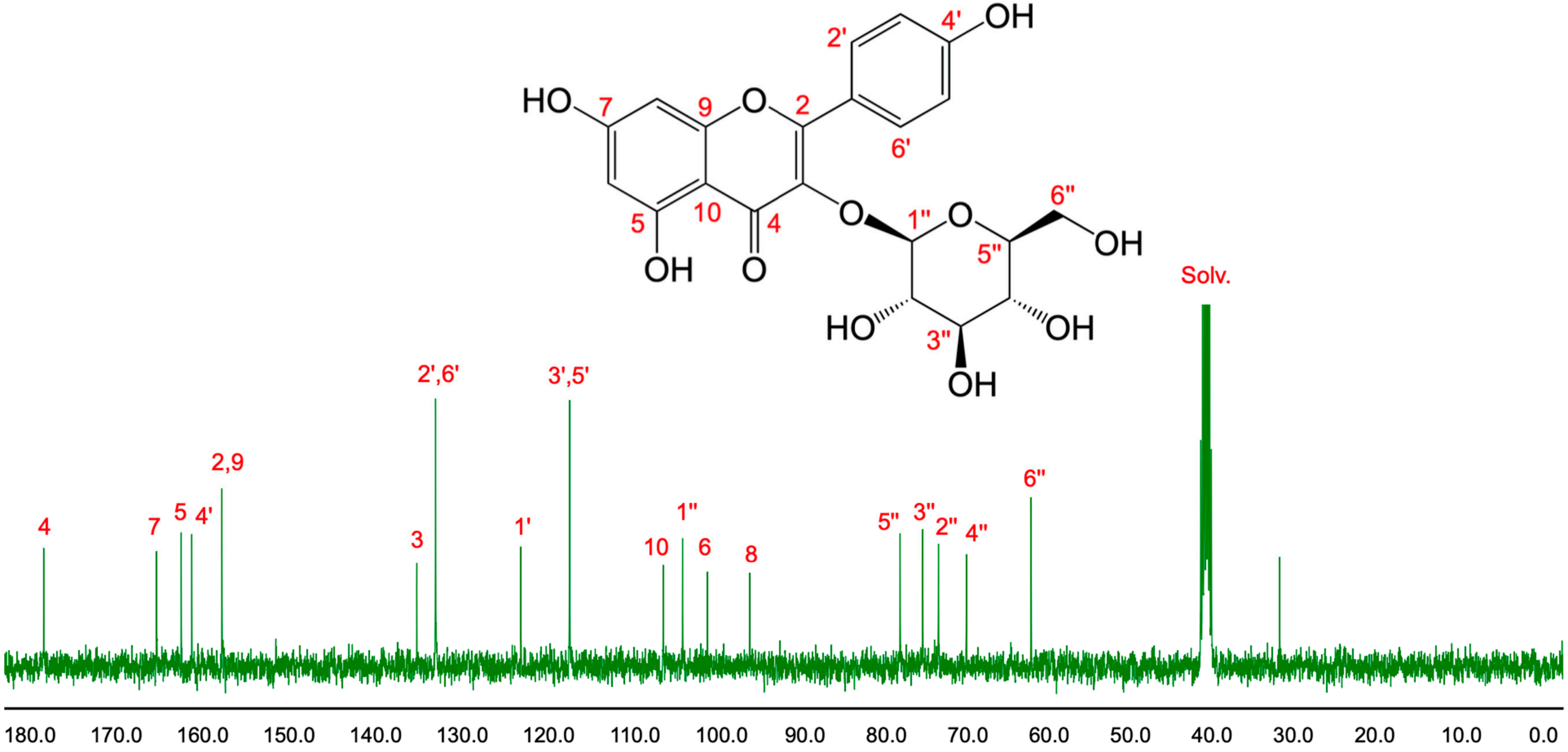

Figure S3. ^{13}C NMR spectrum of trifolin (**2**) (100 MHz, $CDCl_3$).

Table S11. ^{1}H and ^{13}C NMR spectroscopic data of **2** in $CDCl_3$ (δ in ppm, J in Hz).

no.	**2**	
	δ_H	δ_C
1	-	-
2	-	156.9
3	-	133.8
4	-	178.1
5	-	161.7
6	6.17, d, *J* =1.7	99.2
7	-	164.7
8	6.39, d, *J*=2.3	94.2
9	-	156.9
10	-	104.5
1'	-	121.4
2'	8.04, d, *J*=8.6	131.5
3'	6.83, d, *J*=9.2	115.6
4'	-	160.5
5'	6.83, d, *J*=9.2	115.6
6'	8.04, d, *J*=8.6	131.5
1''	5.37, d, *J*=7.5	102.2
2''	-	71.7
3''	-	73.6
4''	-	68.4
5''	-	76.3
6''	3.63, s	60.7
1'''	-	-
2'''	-	-
3'''	-	-
4'''	-	-
5'''	-	-
6'''	-	-

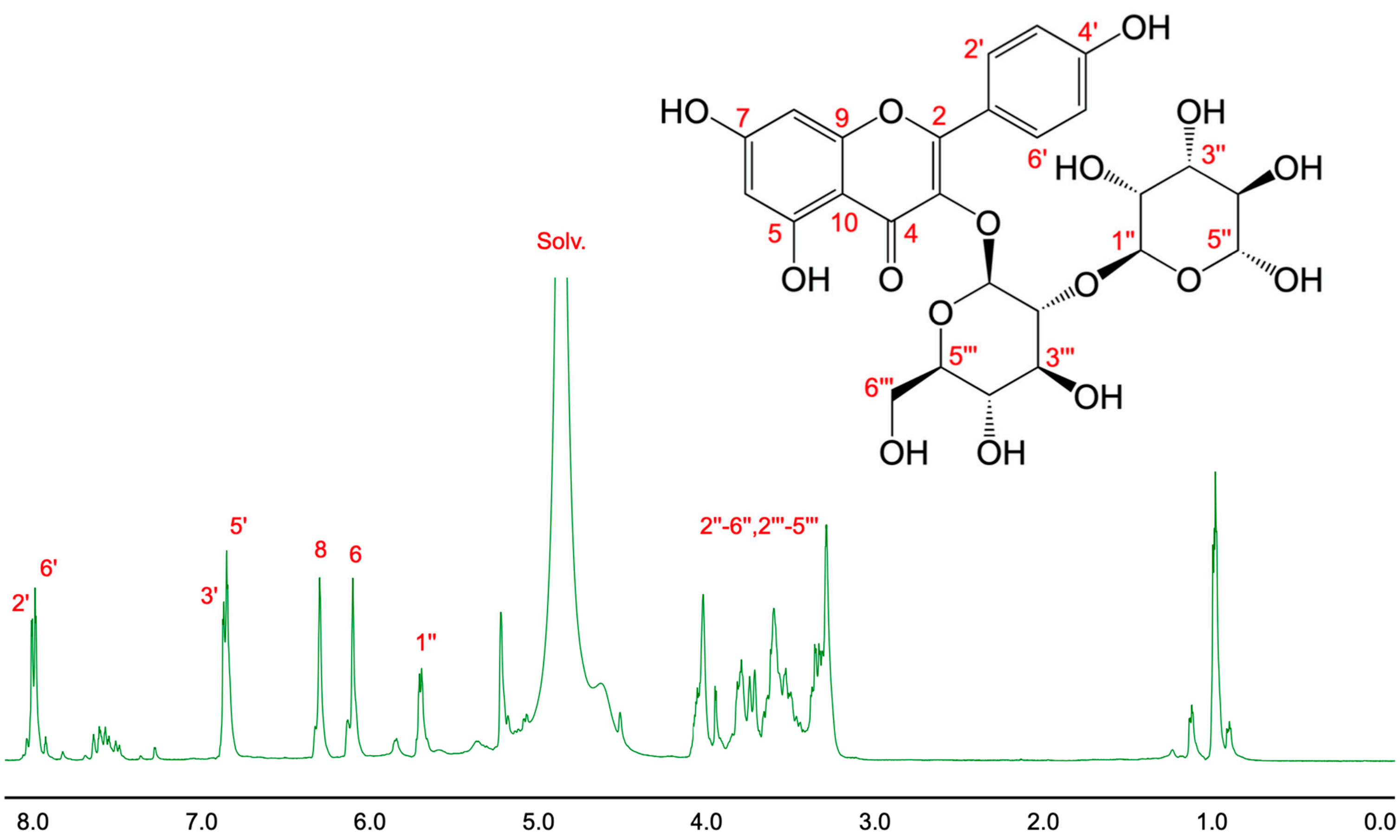

Figure S4. ^{1}H NMR spectrum of kaempferol-3-neohesperidoside (**3**) (400 MHz, CD_3OD).

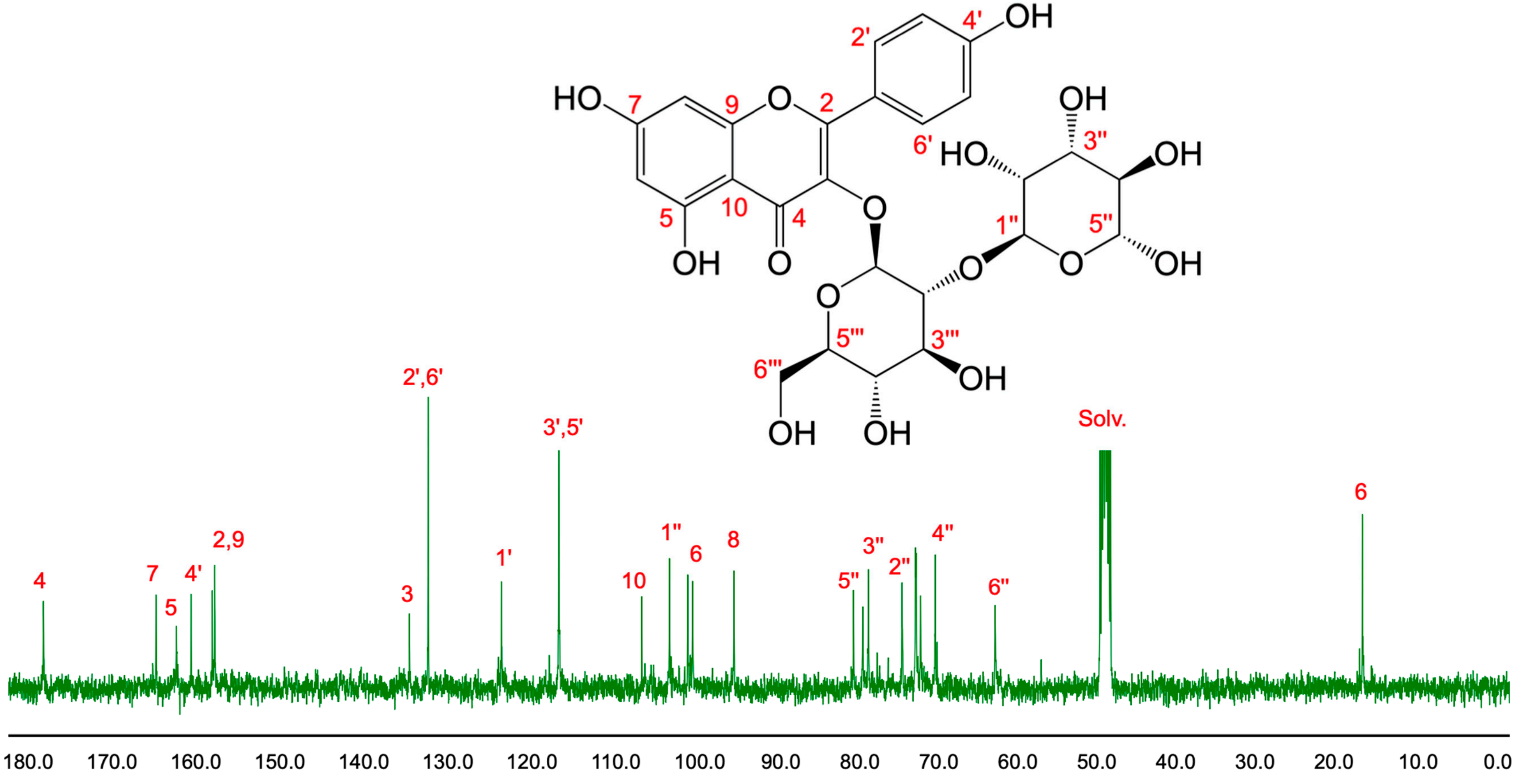

Figure S5. ^{13}C NMR spectrum of kaempferol-3-neohesperidoside (**3**) (100 MHz, CD$_3$OD).

Table S12. ^1H and ^{13}C NMR spectroscopic data of **3** in CD_3OD (*δ* in ppm, *J* in Hz).

no.	3	
	δ_H	δ_C
1	-	-
2	-	159.8
3	-	133.1
4	-	177.9
5	-	161.6
6	6.11, s	98.4
7	-	164.1
8	6.31, s	93.3
9	-	156.9
10	-	104.6
1'	-	121.8
2'	8.01, d, *J*=8.7	130.8
3'	6.87, d, *J*=8.7	114.8
4'	-	157.2
5'	6.87, d, *J*=8.7	114.8
6'	8.01, d, *J*=8.7	130.8
1''	5.71, d, *J*=5.0	98.9
2''	3.4-3.60, m	78.6
3''	3.52, m	77.4
4''	3.29-3.35, m[a]	70.4
5''	3.50-3.53, m	76.8
6''	4.02-4.05, m[b]	61.2
1'''	5.23, br s	101.2
2'''	3.79-3.82, m	71.0
3'''	3.73, d, *J*=12.4	70.9
4'''	3.29-3.35, d, m[a]	72.6
5'''	4.02-4.05, m[b]	68.6
6'''	0.97, d, *J*=3.2	16.2

[a]overlapped, [b]overlapped

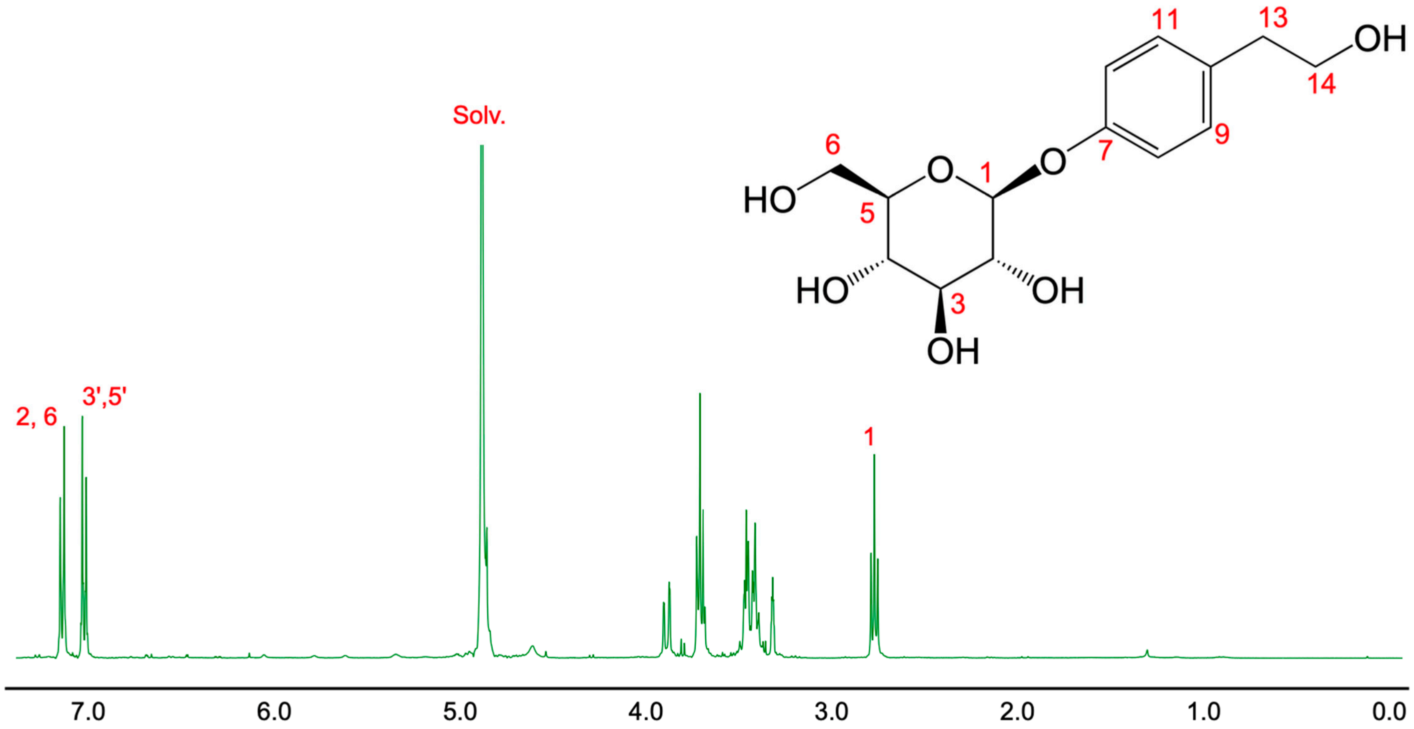

Figure S6. ^{1}H NMR spectrum of icariside D_2 (**4**) (400 MHz, CD_3OD).

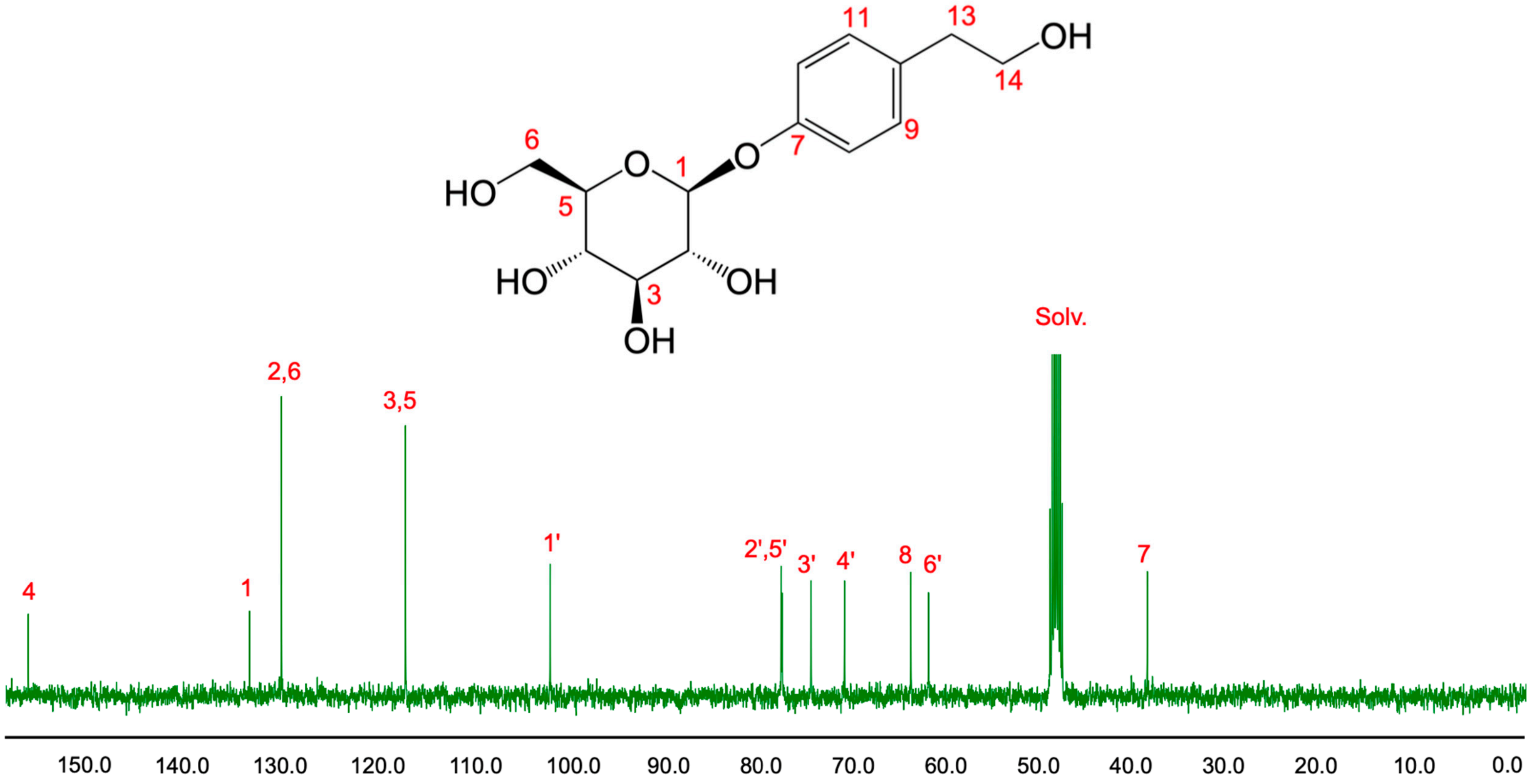

Figure S7. ^{13}C NMR spectrum of icariside D2 (**4**) (100 MHz, CD_3OD).

Table S13. ^{1}H and ^{13}C NMR spectroscopic data of **4** in CD_3OD (δ in ppm, J in Hz).

no.	**4**	
	δ_H	δ_C
1	-	132.9
2	7.12, d, J=8.2	129.6
3	7.00, d, J=8.2	116.5
4	-	156.3
5	7.00, d, J=8.2	116.5
6	7.12, d, J=8.2	129.6
7	2.74, t	38.1
8	3.79, m	63.0
1'	3.86, dd, J=11.91	101.2
2'	3.29, t	73.6
3'	3.39, m	76.6
4'	3.39, m	70.0
5'	3.47, m	76.8
6'	3.70, m	61.2

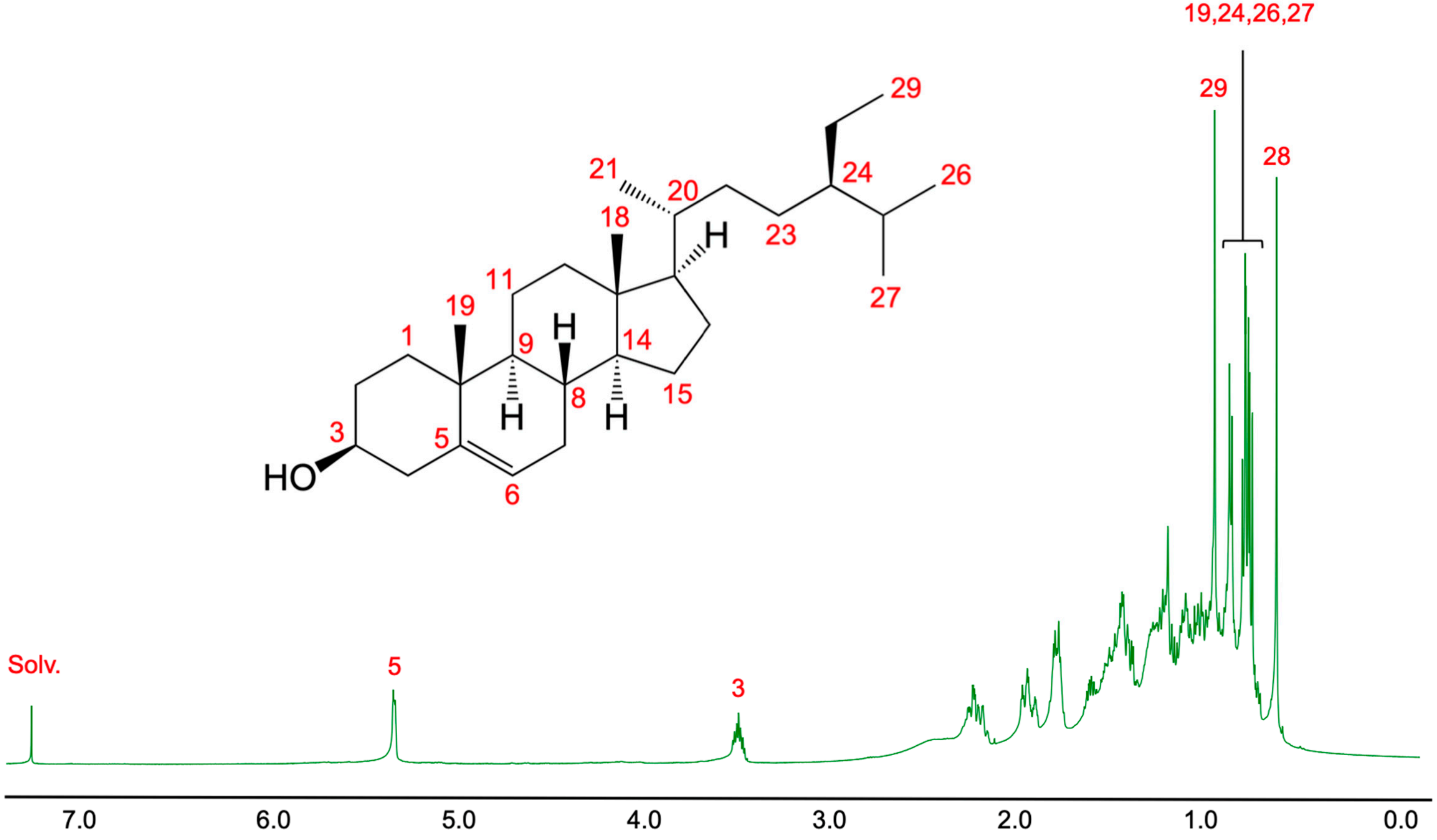

Figure S8. ^{1}H NMR spectrum of β-sitosterol (**5**) (400 MHz, $CDCl_3$).

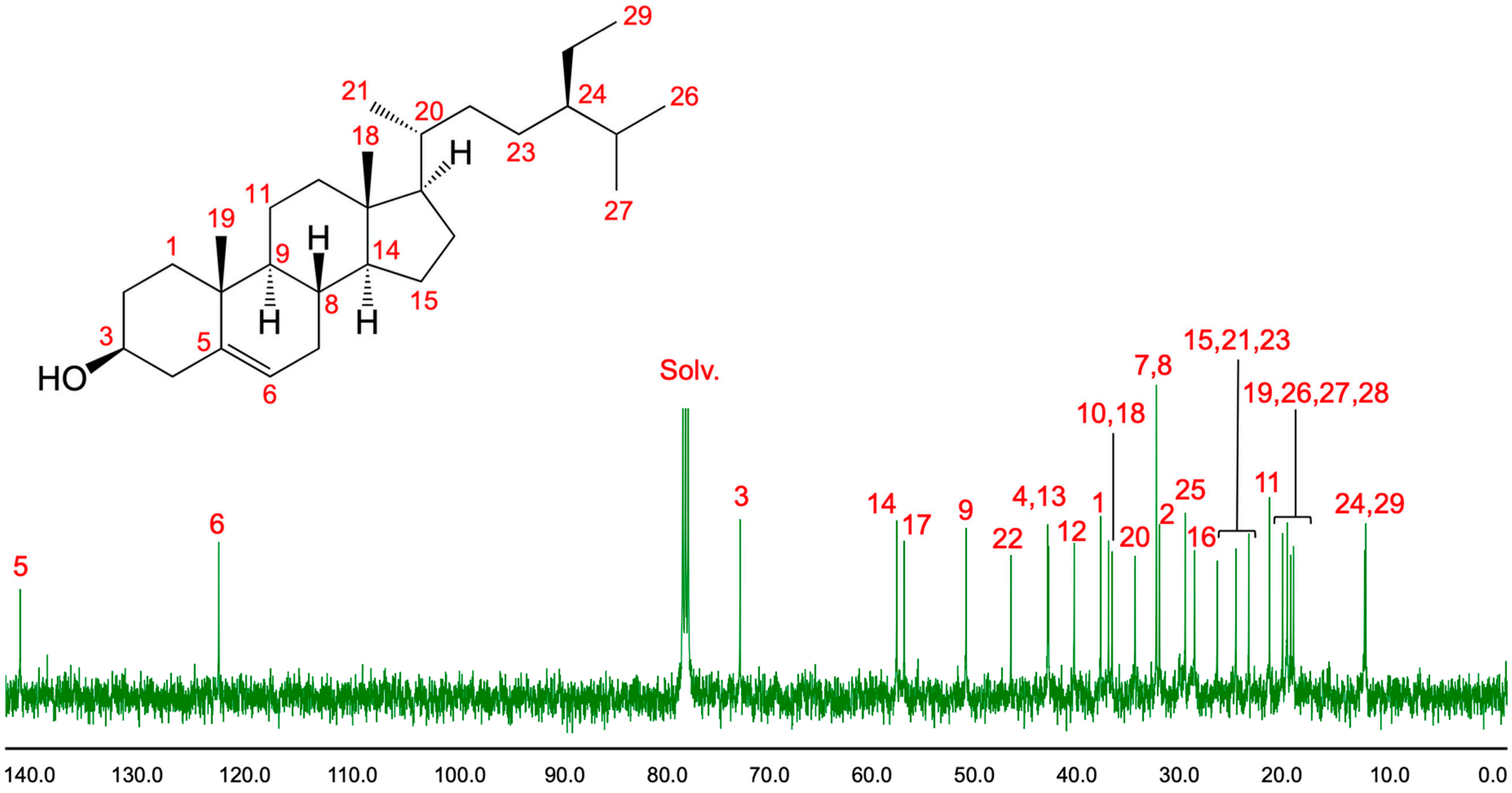

Figure S9. ^{13}C NMR spectrum of β-sitosterol (**5**) (100 MHz, $CDCl_3$).

Table S14. ^1H and ^{13}C NMR spectroscopic data of **5** in $CDCl_3$ (δ in ppm, *J* in Hz).

no.	5	
	-	37.3
1	-	31.7
2	3.53, m	71.9
3	-	42.4
4	5.32, t (4.6)	140.8
5	-	121.8
6	-	31.9
7	-	31.9
8	-	50.2
9	-	36.6
10	-	21.2
11	-	39.9
12	-	42.4
13	-	56.9
14	-	26.2
15	-	28.3
16	-	56.1
17	-	36.2
18	0.90, d, (6.3)	19.1
19	-	34.0
20	-	26.2
21	-	45.9
22	-	23.2
23	0.83, d (7.5)	12.1
24	-	29.2
25	0.82, d (6.9)	19.9
26	0.79, d (6.9)	19.5
27	0.66, s	18.9
28	0.99, s	11.9
29	-	37.3
